# Supplementary material for: Weather-Based Predictive Modeling of Cercospora beticola Infection Events in Sugar Beet in Belgium
Source: J Fungi (Basel). 2021 Sep 18;7(9):777. doi: 10.3390/jof7090777 (PMC8470031; doi:10.3390/jof7090777)
Supplement: Supplementary file 1 [file jof-07-00777-s001.zip › jof-1357917-supplementary.pdf]

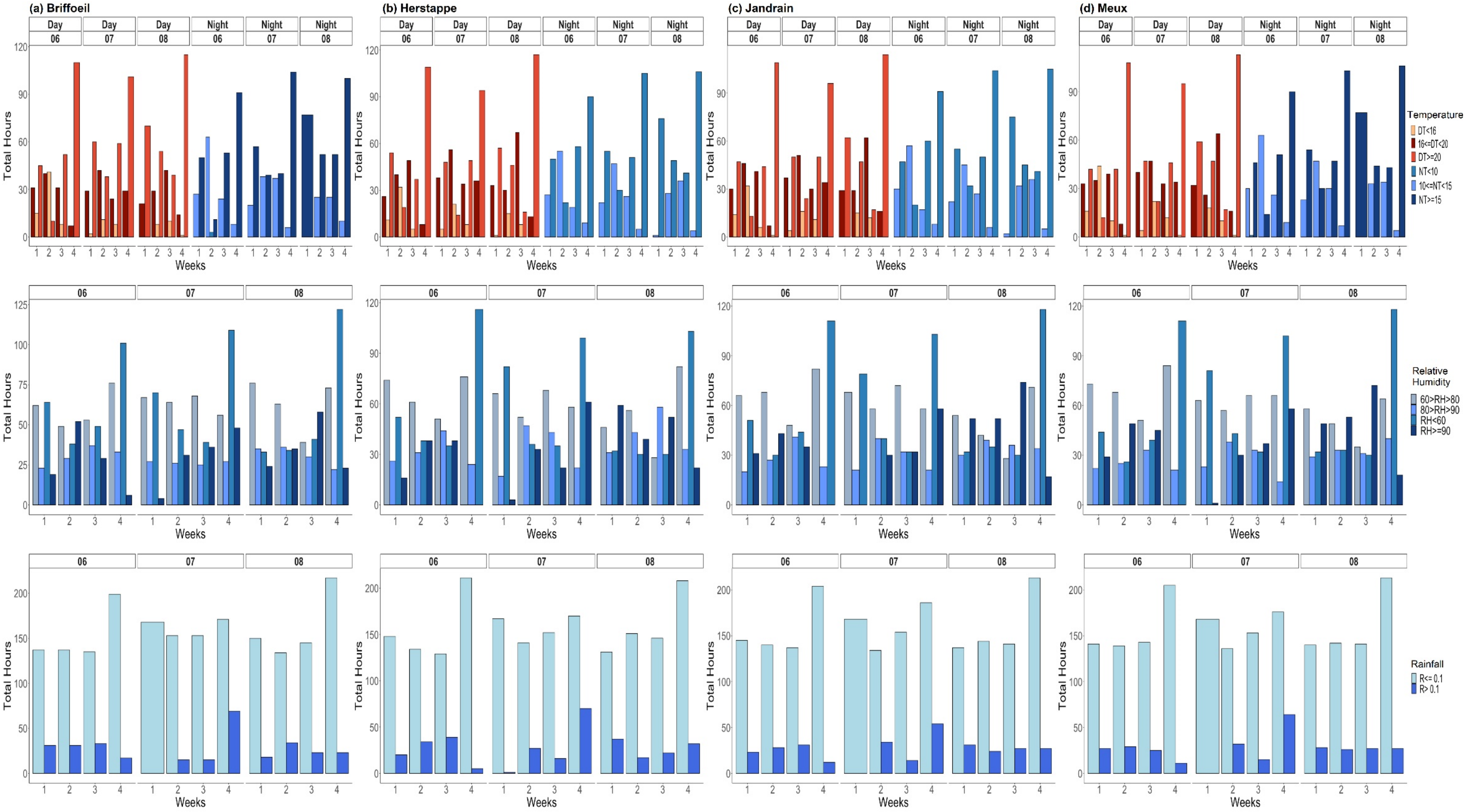

Figure S1. Distribution of weekly total hours for defined ranges of temperature (° C), relative humidity (%) and rainfall (mm) at Briffoeil (a), Herstappe (b), Jandrain (c), and Meux (d), Belgium, during June (06), July (07) and August (08) 2019. DT and NT stand for daytime (7.00 a.m. to 8.59 p.m.) and nighttime (9.00 p.m. to 6.59 a.m. the following day) temperatures, respectively. The intervals of weather vaariables are provided in Table 2. (Note differences of scales on the y-axis.)

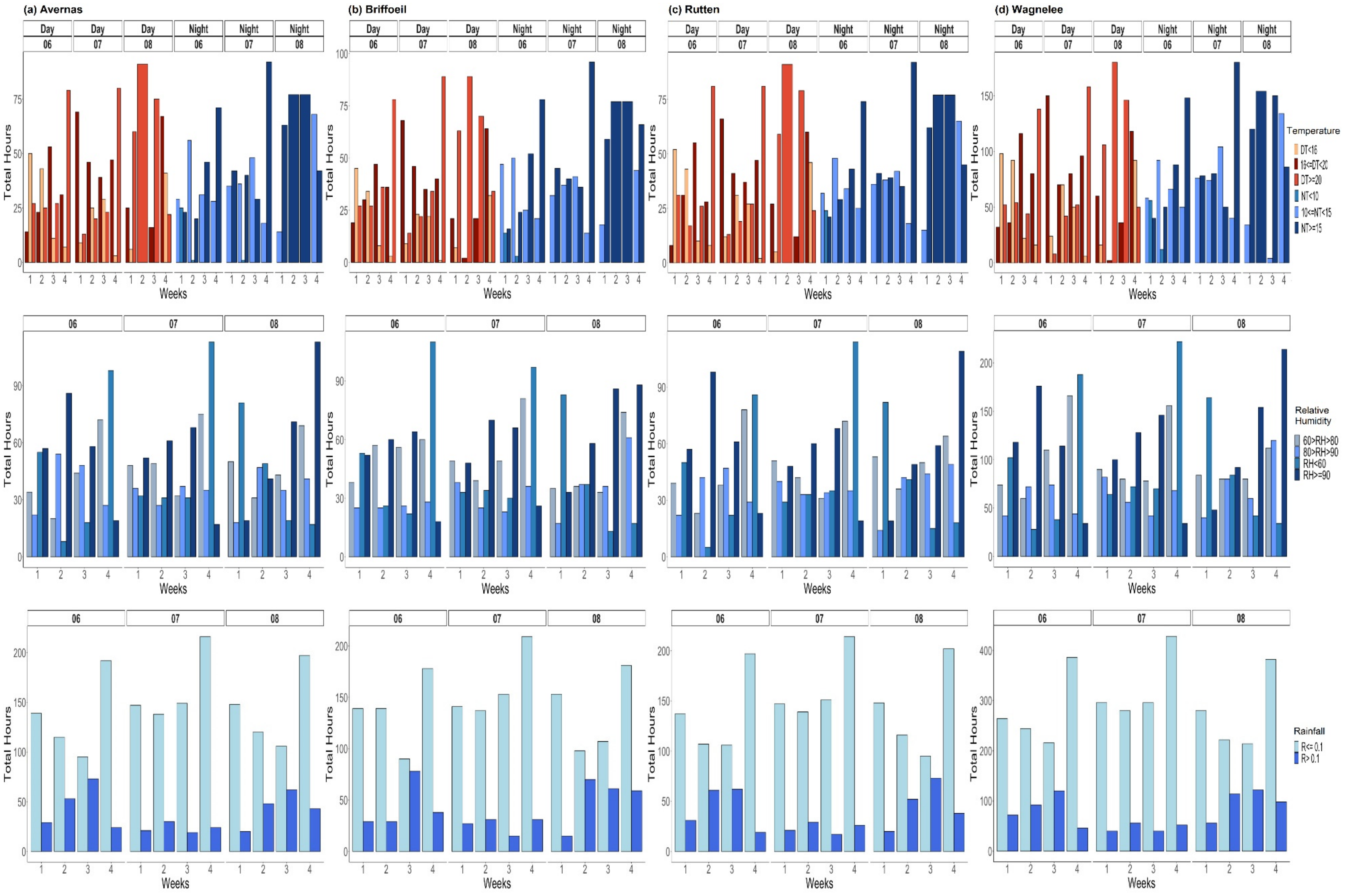

Figure S2. Distribution of weekly total hours for defined ranges of temperature (° C), relative humidity (%) and rainfall (mm) at Avernas (a), Briffoeil (b), Rutten (c), and Wagnelée (d), Belgium, during June (06), July (07) and August (08) 2020. DT and NT stand for daytime (7.00 a.m. to 8.59 p.m.) and nighttime (9.00 p.m. to 6.59 a.m. the following day) temperatures, respectively. The intervals of weather vaariables are provided in Table 2. (Note differences of scales on the y-axis.)

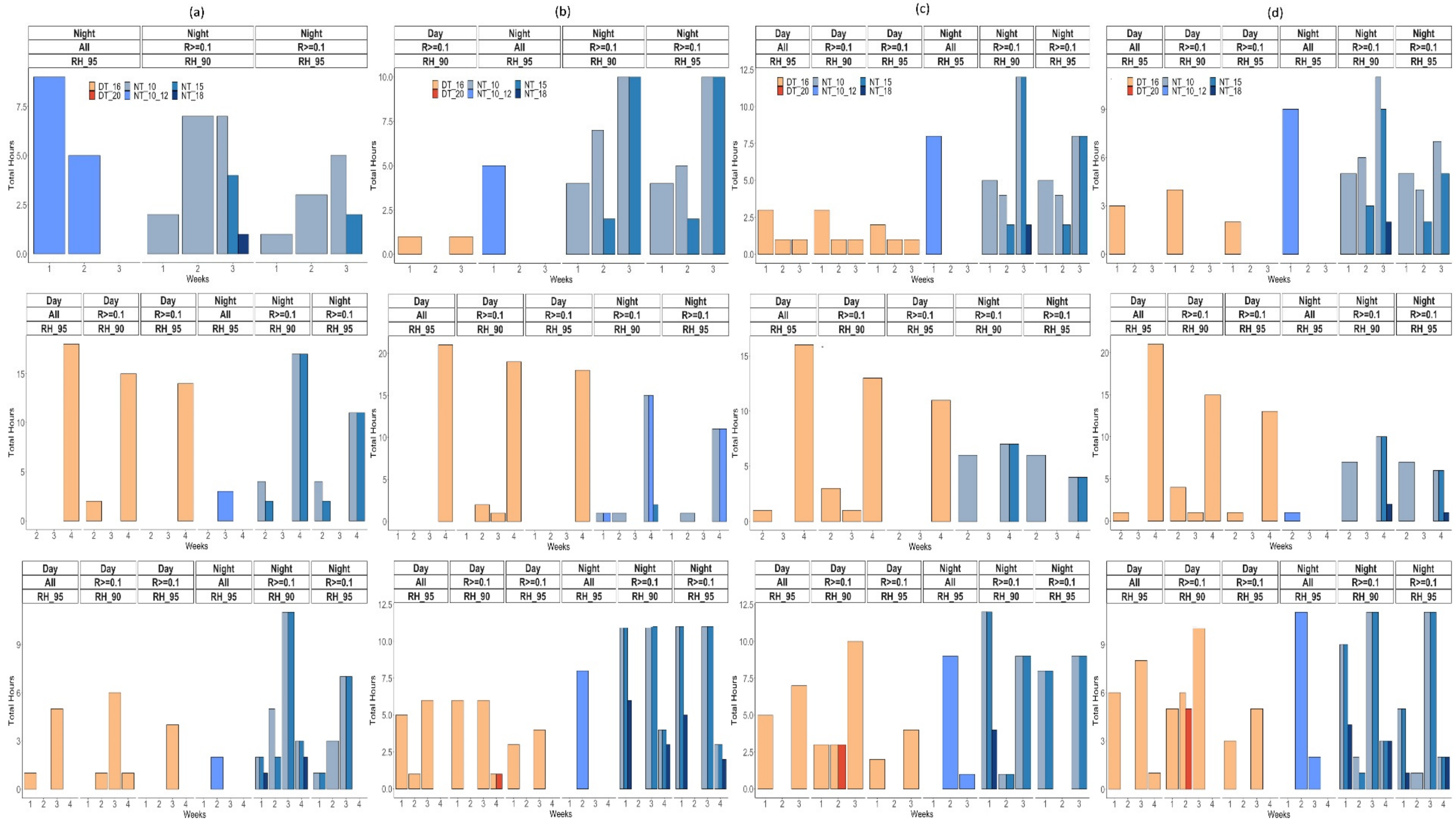

Figure S3. Weekly total hours of weather conditions during the months of June (top) to August (bottom) 2019 at Briffoeil (a), Herstappe (b), Jandrain (c) and Meux (d). Weather conditions are presented as the defined combinations of rainfall (R), relative humidity (RH), and daytime and nighttime temperature (T) being met simultaneously. RH_95: RH ≥95%; RH_90: RH ≥90%; DT_16: daytime T ≥16 °C; DT_20: daytime T ≥20 °C; NT_10: nighttime T ≥10 °C; NT_15: nighttime T ≥15 °C; NT_10_12: nighttime T between 10 °C and 12 °C; NT_18: nighttime T ≥18 °C. Daytime: 7.00 a.m. to 8.59 p.m.; nighttime: 9.00 p.m. to 6.59 a.m. the following day. (Note differences of scales on the y-axis.)

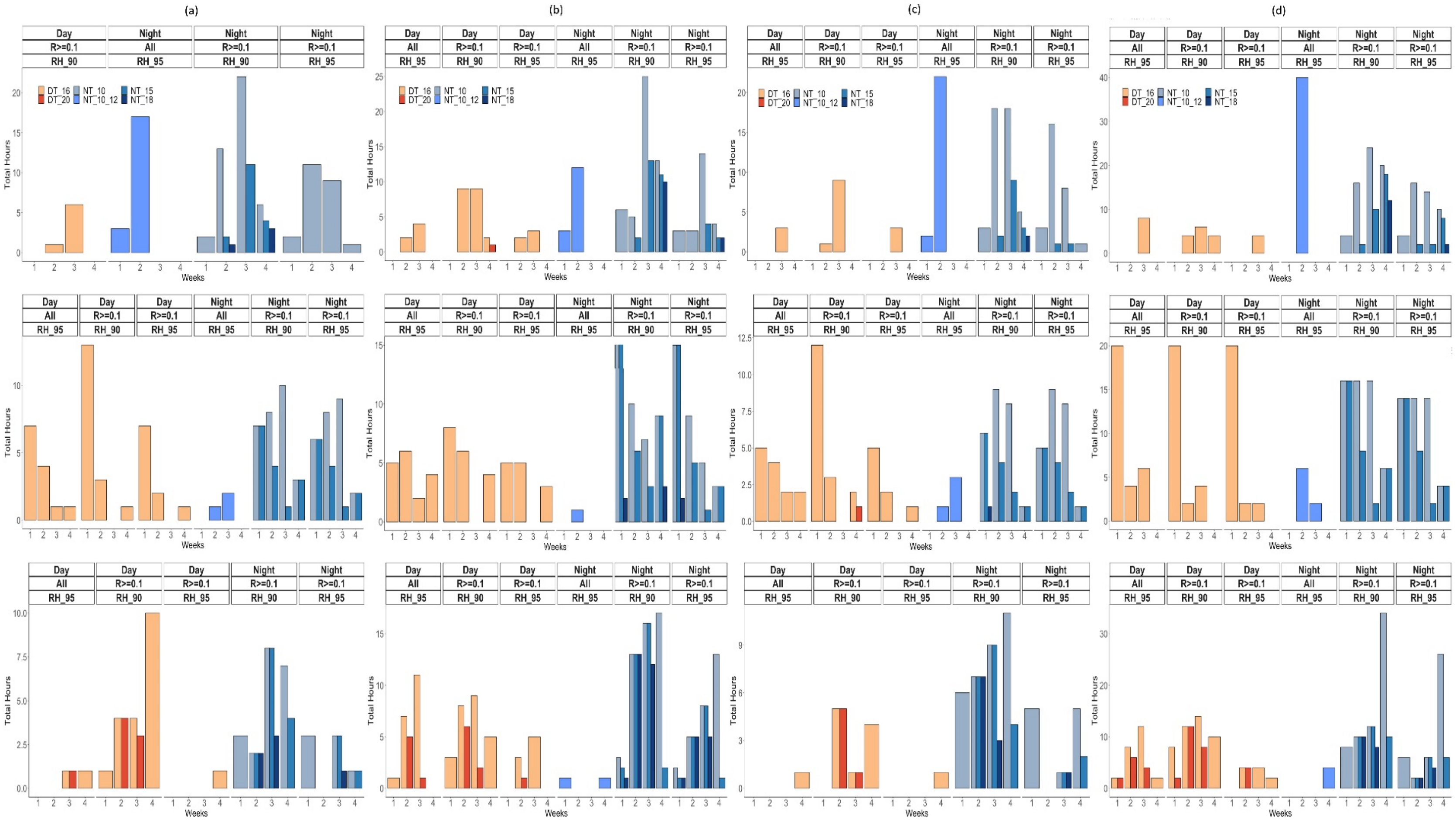

Figure S4. Weekly total hours of weather conditions during the months of June (top) to August (bottom) 2020 at Avernas (a), Briffoeil (b), Rutten (c), and Wagnelée (d). Weather conditions are presented as the defined combinations of rainfall (R), relative humidity (RH), and daytime and nighttime temperature (T) being met simultaneously. RH_95: RH ≥95%; RH_90: RH ≥90%; DT_16: daytime T ≥16 °C; DT_20: daytime T ≥20 °C; NT_10: nighttime T ≥10 °C; NT_15: nighttime T ≥15 °C; NT_10_12: nighttime T between 10 °C and 12 °C; NT_18: nighttime T ≥18 °C. Daytime: 7.00 a.m. to 8.59 p.m.; nighttime: 9.00 p.m. to 6.59 a.m. the following day. (Note differences of scales on the y-axis.).
